# Tunable Pentapeptide Self‐Assembled β‐Sheet Hydrogels

**DOI:** 10.1002/anie.201801001

**Published:** 2018-05-17

**Authors:** David E. Clarke, Christopher D. J. Parmenter, Oren A. Scherman

**Affiliations:** ^1^ Melville Laboratory for Polymer Synthesis Department of Chemistry University of Cambridge Lensfield Road Cambridge CB2 1EW UK; ^2^ Nottingham Nanoscale and Microscale Research Centre University of Nottingham University Park Nottingham NG7 2RD UK

**Keywords:** β-sheet self-assembly, hydrogels, nanostructures, oligopeptides, supramolecular chemistry

## Abstract

Oligopeptide‐based supramolecular hydrogels hold promise in a range of applications. The gelation of these systems is hard to control, with minor alterations in the peptide sequence significantly influencing the self‐assembly process. We explored three pentapeptide sequences with different charge distributions and discovered that they formed robust, pH‐responsive hydrogels. By altering the concentration and charge distribution of the peptide sequence, the stiffness of the hydrogels could be tuned across two orders of magnitude (2–200 kPa). Also, through reassembly of the β‐sheet interactions the hydrogels could self‐heal and they demonstrated shear‐thin behavior. Using spectroscopic and cryo‐imaging techniques, we investigated the relationship between peptide sequence and molecular structure, and how these influence the mechanical properties of the hydrogel. These pentapeptide hydrogels with tunable morphology and mechanical properties have promise in tissue engineering, injectable delivery vectors, and 3D printing applications.

The self‐assembly of oligopeptide sequences into nanostructures holds promise for a range of applications in biomedicine, food science, cosmetics, and nanotechnology.^1^, ^2^, ^3^ These materials can be readily synthesized, providing hydrogel systems with robust mechanical properties.^3^ Experimental and computational approaches have yielded a selection of di‐ and tripeptide sequences,^3^, ^4^, ^5^, ^6^, ^7^ which have been proven to assemble into nanostructures and hydrogels under aqueous conditions, generating nanospheres,^8^ fibrous and plate‐like assemblies,^9^, ^10^ heterogeneous nanostructures,^4^, ^11^, ^12^ and micelles and nanotubes.^13^, ^14^, ^15^ To improve gelation characteristics, these small molecules often require either the inclusion of aromatic amino acid residues or a synthetic terminal group.^1^, ^16^, ^17^, ^18^, ^19^ This introduces π–π stacking and hydrophobic interactions, which promote self‐assembly and gelation.^3^ However, synthetic terminal groups are not inherently biodegradable and are therefore less likely to be suitable for biological applications. Additionally, minor alterations in the sequence can significantly influence the self‐ assembly process, which makes both design and further functionalization difficult, whereby typical self‐assembly rules cannot be applied.

The native tripeptide sequences discovered to self‐assemble into stable hydrogels have contained aromatic amino acids such as the KYF and DFY motifs.^3^, ^20^ Oligopeptides that consist of amino acids with aliphatic side chains have received less attention.^21^, ^22^ Furthermore, outside of tripeptide assemblies, there have only been a few studies which focused on oligopeptide sequences that are slightly extended in length (4–8 amino acids). In a few cases, these studies have been based on short peptide fragments of larger polypeptides already known to self‐assemble into nanostructures, such as NFGAIL^5^, ^23^ (fragment of human islet polypeptide) and KLVFFAE^24^ (part of amyloid β_16–22_). Most recently, Pappas et al. utilized a dynamic combinatorial peptide library with dipeptide inputs and discovered that sequences of four residues (W4, F2L2) and six residues (F6, L6) formed higher‐order assemblies. Additionally, the eight‐residue FDFSFDFS sequence was also able to form a self‐supporting hydrogel.^22^

We hypothesized that exploring the self‐assembly of pentapeptides would provide flexibility in chemical design and gelation propensity, while allowing for simplicity in synthesis for future applications. We report three pentapeptide sequences that are free of aromatic groups and can form highly robust hydrogels with stiffnesses spanning two orders of magnitude from 2 to 200 kPa (Figure 1). The peptide sequences discovered were found to contain three aliphatic isoleucine (Ile) residues, an amino acid with a high propensity to form β‐sheets.^25^, ^26^ These aliphatic amino acids were further combined with two aspartic acid (Asp) residues, which improve the solubility of hydrophobic Ile. Then, upon protonation, charge recognition/hydrogen bonding drives β‐sheet self‐assembly and hydrogel formation. To further investigate the self‐assembly of the pentapeptide sequences, the positions of the charged Asp residues were systematically altered to generate three different charge distributions (Figure 1): Asp flanking a central Ile region (DI3D), Asp at the N‐terminus of the sequence (D2I3), and Asp alternating with Ile residues (IDIDI). Using these three sequences and their different architectures, we aimed to explore the relationship between amino acid sequence and molecular structure, and their influence on the mechanical properties of the hydrogel.

**Figure 1:**
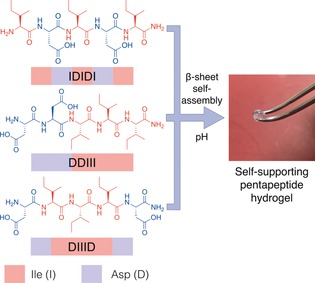
Structures of the three pentapeptide sequences, which can form robust hydrogels (left), and image of the D2I3 peptide hydrogel (2 wt %) being held with tweezers (right).

In an initial screen, we tested different peptide designs and sequence lengths, which yielded differences in solubility and gelation. These included an additional pentapeptide sequence (DI4), a tetrapeptide (DI2D), and a valine variant (DV3D). The DI4 sequence was not soluble in aqueous media and could not be purified. The DI2D and DV3D sequences could be solubilized in aqueous media, but no obvious self‐assembly or gel formation was witnessed. From this initial screen, a ratio of 2 Asp to 3 Ile within a pentapeptide sequence proved most successful, enabling both purification of the peptides and subsequent assembly into robust hydrogels.

Peptide stock solutions were dissolved at 1 and 2 wt % in a basic aqueous media at pH 10 through sonication. These stock solutions were then aliquoted onto a hydrophobic surface and a small volume of HCl pipetted onto each droplet to achieve a neutral pH. Upon the addition of HCl, the peptide solution gelled and could be manipulated with tweezers (Figure 1).

The mechanical properties of the hydrogels were studied by oscillatory shear rheology. Hydrogel formation was verified, as the storage modulus (G′) exceeded the loss modulus (G′′) at both 1 and 2 wt % (see Figures S2 and S3 in the Supporting Information). The frequency sweeps show that the mechanical properties of all the hydrogels were independent of oscillation frequency, and this is consistent across the three sequences studied (see Figures S2 A and S3 A). The hydrogels were also evaluated under the application of shear strain. The moduli remained in the linear elastic region up to strains of around 1 % with little change in G′, followed by a significant decrease in G′ for strains exceeding 2 % (see Figures S2 B and S3 B).

The stiffness of the hydrogels was dependent on both hydrogel concentration and the charge distribution of the peptide sequence (Figure 2 A). At both 1 and 2 wt %, the D2I3 sequences generated the stiffest gels, and under the same conditions the IDIDI hydrogels exhibited the lowest G′ values. Comparing IDIDI (1 wt %) and D2I3 (2 wt %) hydrogels, the G′ value increased by two orders of magnitude from 2 to 200 kPa, respectively. These stiffness values are in the region of many soft tissues and compare well to previously published peptide hydrogel systems, including aromatic peptides^4^, ^17^ and peptide–amphiphile hydrogels.^27^, ^28^ The ability to tune the G′ value across a large range holds great promise for applications in tissue engineering, given that the behavior of cells has been found to be heavily influenced by the mechanical properties of their surrounding environment.^29^, ^30^

**Figure 2:**
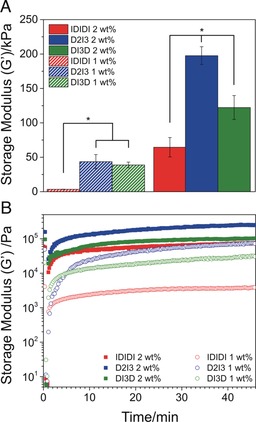
A) Storage moduli taken from frequency sweeps at 0.1 % strain. Hydrogel stiffness can be controlled by both concentration and the charge distribution of the peptide sequence. Error bars represent ±standard deviation and *p<0.05. B) Sequential step strain sweeps: 0.1 % strain (0–30 s), 200 % strain (30–60 s), followed by a 45 min recovery period (0.1 % strain); all steps were performed at an oscillation frequency of 6.283 rad s^−1^ and demonstrate that all gels were able to recover their mechanical properties after failure.

One of the primary benefits of using noncovalent interactions is their ability to reform after deformation, allowing self‐assembled hydrogels to recover their mechanical properties after the application of high strains.^31^, ^32^ To investigate the self‐healing performance of these systems, a series of step strain measurements were carried out (Figure 2 B). All the hydrogels displayed a steep incline in modulus, recovering around 50 % of G′ within 5 min, followed by a plateau and complete recovery between 10 and 20 min. These self‐healing properties can also be cycled (see Figure S4). The ability to repeatedly recover mechanical properties highlights the dynamic nature of these hydrogels, in which the β‐sheets can adopt more energetically favorable and mechanically robust conformations over time.^32^ The dynamic nature of these systems is further supported by their shear‐thinning characteristics, which were evaluated using flow sweeps (see Figure S5 A,B). All the hydrogels displayed typical shear‐thinning behavior with viscosity decreasing linearly with increasing shear stress. The combination of both self‐healing and shear‐thinning capabilities renders these hydrogels ideal for biomedical applications that require recovery after significant deformation, such as injectable therapies or 3D printing.

To investigate the relationship between supramolecular structure and mechanical properties, the secondary structure of the peptide assemblies in the hydrogels were studied using spectroscopic techniques. The CD spectra of the hydrogels resembled a β‐sheet, with a minimum between 220 and 230 nm (Figure 3 A; see also Figures S5 A and S6 A). This structure was supported by the amide I region of FTIR spectra (see Figures S6 B and S7 B), in which all hydrogels displayed a prominant peak at 1630 cm^−1^ indicating a β‐sheet conformation.^33^, ^34^ However, differences in the CD and FTIR spectra were evident for each of the sequences studied. The CD spectra differed in intensity and were red‐shifted relative to those of model β‐sheets, which typically have a maximum at 195 nm and a minimum at 216 nm.^34^

**Figure 3:**
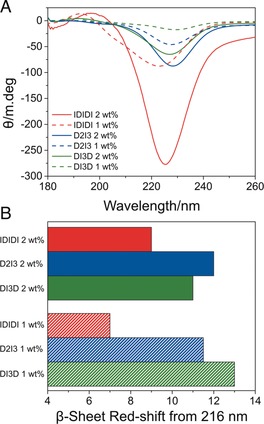
A) Circular dichroism of the pentapeptide hydrogels at 1 and 2 wt %. The minimum between 220 and 230 nm is typically indicative of β‐sheet formation. B) β‐Sheet red‐shift from 216 nm taken from the circular dichroism spectra.

The CD signatures of β‐sheets are known to have greater variability than those of other peptide secondary structures.^28^ β‐Sheets have both significant intermolecular and intrastrand hydrogen bonding.^35^ Furthermore, peptides can form antiparallel, parallel, or mixed β‐sheets, which will influence both the strands in the assemblies as well as the networks they form.^28^ We analyzed the relative red‐shifts in the CD minima of the different hydrogels. The IDIDI sequence provided the softest gels and had the smallest red‐shift at both 1 and 2 wt % (Figure 3 A,B). In contrast, DI3D and D2I3 materials had similar red‐shifts with no significant difference in G′ at 1 wt %. However, at 2 wt % the D2I3 sequence was significantly stiffer and had the greatest red‐shift in the CD spectra at this concentration (Figure 3 A,B). Previous studies have suggested that a red‐shift in the CD spectra of β‐sheets is representative of more twisted and distorted arrangements.^28^, ^36^, ^37^ The degree of twisting of β‐sheets is centered around the middle of the sequence.^34^ In twisted β‐sheets, the hydrogen‐bonding distance increases as the angle between two peptides increases, weakening the intermolecular forces and hydrogen bonds on the periphery of the β‐sheet.^38^, ^39^ This will influence the intermolecular forces between individual peptide sequences in the β‐sheet and the morphology of the structures present in the hydrogel.^35^ A difference in β‐sheet peak intensity at 220–230 nm was also observed. The CD measurements were performed at the concentration found in the hydrogel, and in some cases the hydrogels were partially opaque, which is likely to result in some fraction of the light being scattered, influencing peak intensity.

The morphology of the different hydrogels was characterized by cryo‐focused ion beam scanning electron microscopy (cryo‐FIB SEM). In this technique, hydrogel samples are plunged into liquid ethane, rapidly freezing the water content to obtain a thin layer of vitreous ice. This preserves the morphology of the structure in aqueous solution and eliminates drying effects that can be generated when using other preparation techniques. A focused ion beam (FIB) of gallium ions is then used to mill a cross‐section in the sample with an exposed featureless face. Raising the temperature of the stage to 100 °C causes water to slowly sublime away from this face, revealing the underlying physical structure (see Figure S10). This technique allows for imaging of the hydrogels in their native state and in the presence of bound water, overcoming major artefacts associated with drying and water removal (more details of this technique can be found in the Supporting Information).^40^

From the electron micrographs collected, it is evident that the charge distribution in the peptide sequence influences the microstructures of the hydrogels (Figures 4; see also Figures S8 and S9). The IDIDI hydrogels are comprised of high‐aspect‐ratio nanofibers, which at 2 wt % are several microns in length, extending to the height of the trench milled by the FIB (Figure 4 A). At a lower concentration (1 wt %), the IDIDI hydrogels still maintain the same nanofibrillar morphology, but the fibers are shorter in length (Figure 4 B). In comparison, both the D2I3 and DI3D sequences have more entangled microstructures. The D2I3 materials are formed from platelike assemblies interconnected by some fibrous domains (Figure 4 C,D); these observations were further supported by cryo‐transmission electron microscopy images of the D2I3 hydrogels at 2 wt % (see Figure S11). Similarly, the DI3D hydrogels are comprised of some nanofibers but mostly contain dense regions of fibrous bundles (Figure 4 E,F). In summary, it can be observed that the more entangled structures have a greater degree of interconnectivity between the assemblies.

**Figure 4:**
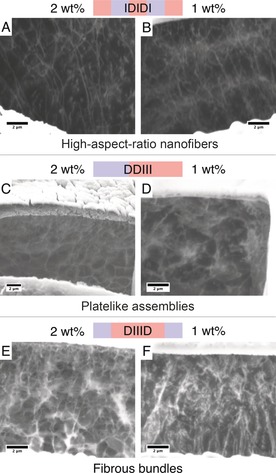
Cryo‐FIB scanning electron micrographs of the hydrogels. A) IDIDI, 2 wt %; B) IDIDI, 1 wt %; C) D2I3, 2 wt % (note: reduced magnification); D) D2I3, 1 wt %; E) DI3D, 2 wt %; and F) DI3D, 1 wt %. The scale bar for all images is 2 μm.

Recently, it has been reported that Asp positioning can influence the stacking orientation of tripeptide β‐sheet assemblies.^20^ Shifting the Asp from the C‐ to the N‐terminus was shown to invert the conformation from a parallel to an antiparallel β‐sheet.^20^ Similarly, both the D2I3 and DI3D peptides contain charged Asp species situated at the termini of the sequence with regions of three repeat Ile residues. Previous studies on polyisoleucines reported that sequential Ile‐rich structures are more stable in twisted parallel β‐sheet arrangements.^35^, ^41^ The FTIR spectra for the D2I3 and DI3D sequences had two minor peaks at 1655 and 1675 cm^−1^ (see Figures S6 B and S7 B). It has been shown that twisted β‐sheets (both in parallel and antiparallel conformations) can display an amide I splitting with a peak between 1680 and 1690 cm^−1^ as well as a peak at 1650 cm^−1^.^28^, ^34^ Although the D2I3 and DI3D sequences in this study cannot be explicitly defined as being in an antiparallel or a parallel orientation, these observations are in agreement with the red‐shifted CD spectra found, which suggests that both the D2I3 and DI3D hydrogels contain more twisted β‐sheets.

The terminal charged groups coupled with weakened hydrogen bonds on the periphery of the D2I3 and DI3D β‐strands will result in a greater potential to form ionic interactions and further hydrogen bonds with other neighboring strands. These interactions will give rise to the entangled and interconnected assemblies attributed to the D2I3 and DI3D hydrogels (Figures 4 C–F; see also Figure S11). In the IDIDI sequence, the Asp residues are positioned more centrally with singular β‐sheet‐forming amino acids (Ile) in the middle and at the termini. Given that this arrangement does not contain a series of repeat Ile residues, it is likely to provide less twisted β‐sheets. These types of structure will have less entropy and disorder, with hydrogen bonds between sequences being equal in length across the peptide chain, which is likely to facilitate planar stacking arrangements and result in the high‐aspect‐ratio nanofiber assemblies in Figure 4 A,B.

The different types of intermolecular interactions and the high‐order assemblies they form influence the mechanical properties of the pentapeptide hydrogels systems. Larger platelike domains that are more interconnected/entangled provided the stiffest hydrogels, whereas the high‐aspect‐ratio fibers in the IDIDI hydrogels behave like discrete structures with little entanglement between neighboring fibers, resulting in softer hydrogels. Furthermore, the 1 wt % IDIDI hydrogels with shorter fiber lengths have less surface area for entanglement, which corresponded with an order‐of‐magnitude decrease in G′ from 60 to 2 kPa. These three different peptide designs demonstrate that alteration of the position of the β‐sheet‐forming amino acids and charge distribution of the sequence serves as a unique approach to control the morphology and tune the mechanical properties of the resultant hydrogel. Both substrate stiffness and substrate shape have been shown to influence cellular behavior.^29^, ^30^, ^42^ Therefore, with control over both of these parameters, the hydrogels have potential to act as tissue‐engineering scaffolds and matrices.

We have reported three pentapeptide sequences free of aromatic groups, which can form robust hydrogels with gelation induced through changes in pH. We demonstrated that the stiffness of the hydrogels can be tuned across two orders of magnitude (2–200 kPa) by altering the concentration and charge distribution of the peptide sequence. Formed through noncovalent interactions, the hydrogels showed self‐healing and shear‐thinning behavior through reassembly of the physical cross‐links. To explore the relationship between molecular design and the mechanical properties of the resulting hydrogel, we utilized spectroscopic techniques, which verified the β‐sheet structure. Depending on the peptide sequence and its charge distribution, different degrees of red‐shift were evident in the CD spectra, which corresponded to the different morphologies of the self‐assembled structures within the hydrogels. Cryo‐FIB SEM indicated that the IDIDI hydrogels were formed from high‐aspect‐ratio nanofibers. In contrast, the D2I3 and DI3D hydrogels had more entangled and interconnected structures, resulting in the stiffest hydrogels. These pentapeptide self‐assembled hydrogels with tunable morphology and mechanical properties, as well as self‐healing and shear‐thinning characteristics, provide a promising platform for tissue engineering, injectable delivery vectors, and 3D printing applications.

## Conflict of interest

The authors declare no conflict of interest.

## Supporting information

As a service to our authors and readers, this journal provides supporting information supplied by the authors. Such materials are peer reviewed and may be re‐organized for online delivery, but are not copy‐edited or typeset. Technical support issues arising from supporting information (other than missing files) should be addressed to the authors.

Supplementary
