# Supplementary material for: Tunable Pentapeptide Self‐Assembled β‐Sheet Hydrogels
Source: Angew Chem Int Ed Engl. 2018 May 17;57(26):7709–13. doi: 10.1002/anie.201801001 (PMC6055752; doi:10.1002/anie.201801001)
Supplement: Supplementary file 1 — Supplementary [file ANIE-57-7709-s001.pdf]

Angewandte Chemie
Eine Zeitschrift der Gesellschaft Deutscher Chemiker

Supporting Information

Tunable Pentapeptide Self-Assembled β-Sheet Hydrogels

*David E. Clarke, Christopher D. J. Parmenter, and Oren A. Scherman**

anie_201801001_sm_miscellaneous_information.pdf

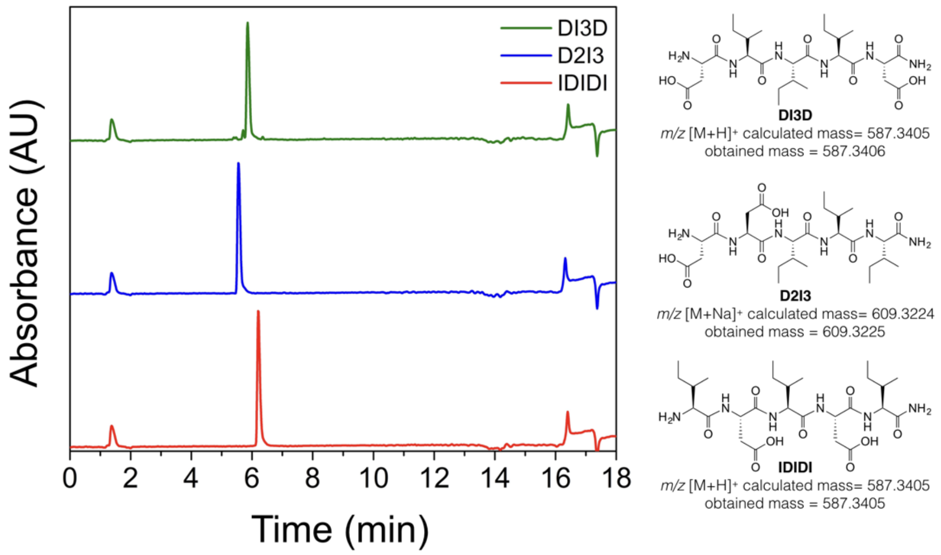

Figure S1. Analytical HPLC traces and corresponding accurate mass (ESI-MS) of the different pentapeptides following purification.

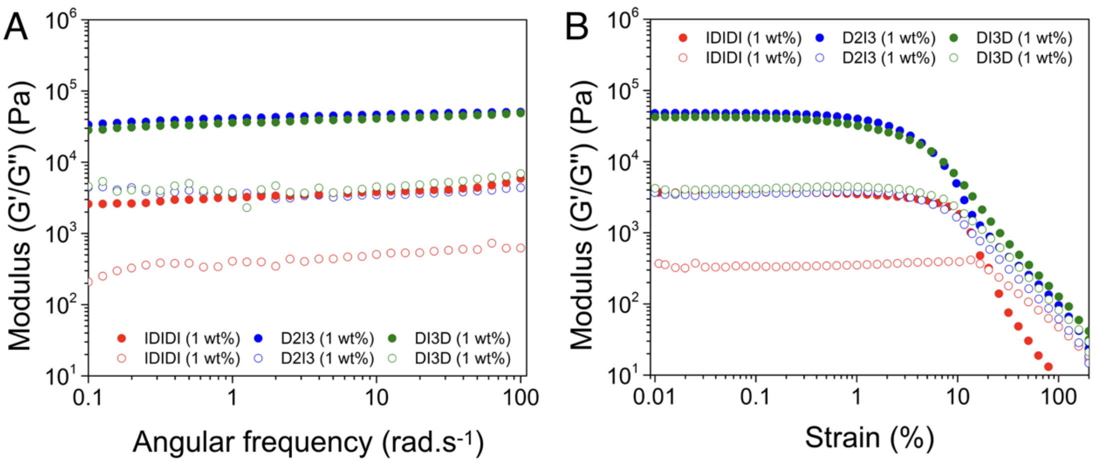

Figure S2. Rheology of hydrogels at 1 wt%. A) Frequency sweep at 0.1% strain, and B) Strain sweep at an oscillation frequency of 6.283 rad s^{-1}.

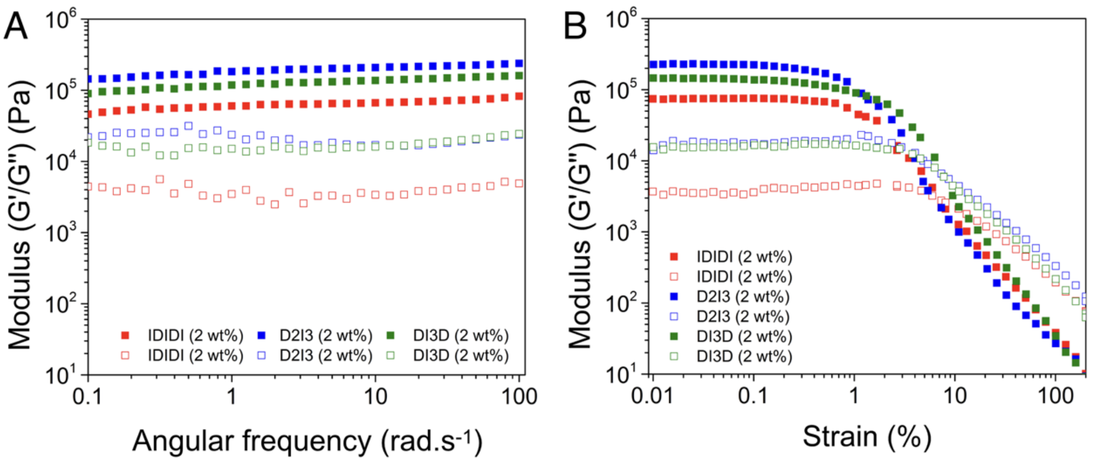

Figure S3. Rheology of hydrogels at 2 wt%. A) Frequency sweep at 0.1% strain, and B) Strain sweep at an oscillation frequency of 6.283 rad s^{-1}.

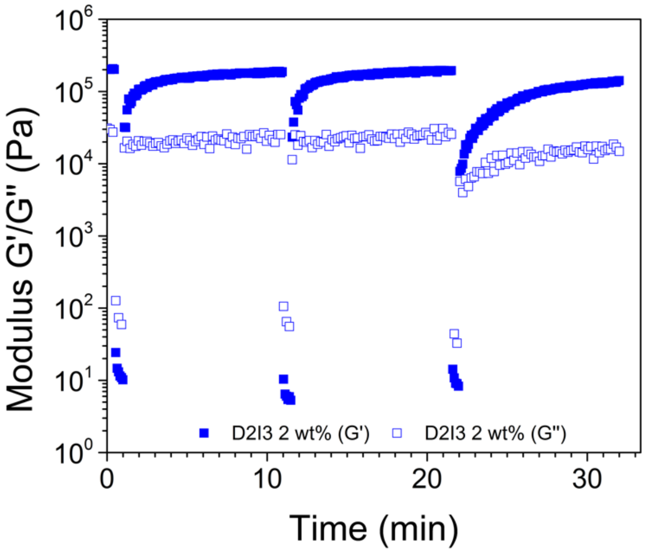

Figure S4. Three repeat step strain sweeps of 0.1% strain (0-30 s), 200% strain (30-60 s) followed by a 10 min recovery period (0.1% strain), all step strains were performed at an oscillation frequency of 6.283 rad s^{-1} and demonstrate that the hydrogels are able to recover their mechanical properties after repeated failure.

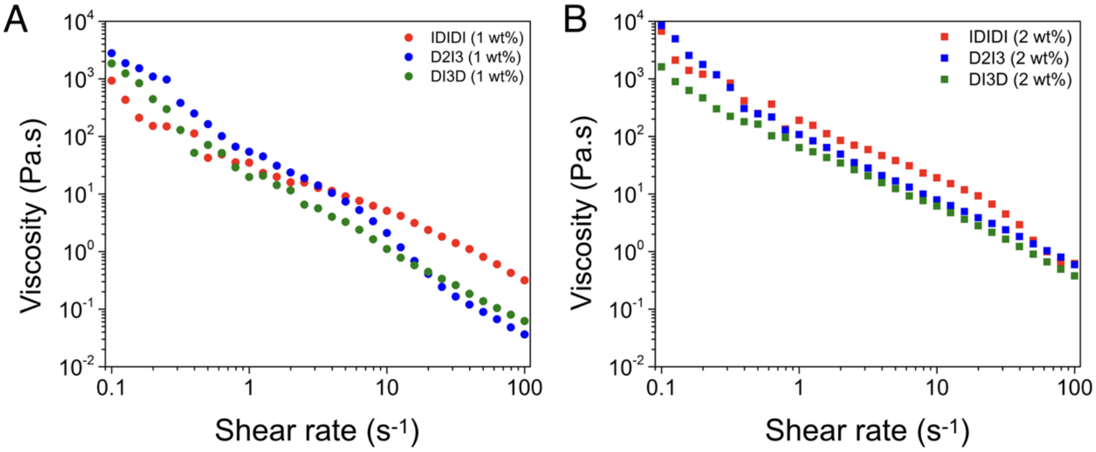

Figure S5. Flow sweeps of hydrogels (5 s equilibration time and 30 s averaging time) at 1 wt% (A) and 2 wt% (B). Viscosity decreasing linearly with increasing shear stress demonstrates shear thinning capability.

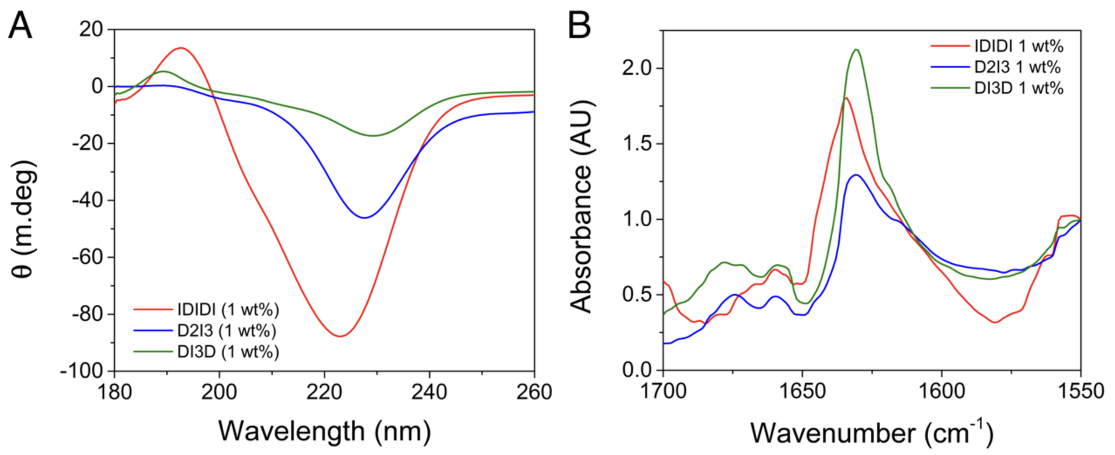

Figure S6. Spectroscopic studies of the pentapeptide hydrogels at 1 wt%. A) Circular dichroism showing minima for hydrogels between 220 nm and 230 nm, typically indicative of β-sheet formation B) FTIR spectra of the amide I region, with a peak at 1630 cm^{-1} indicating β-sheet formation.

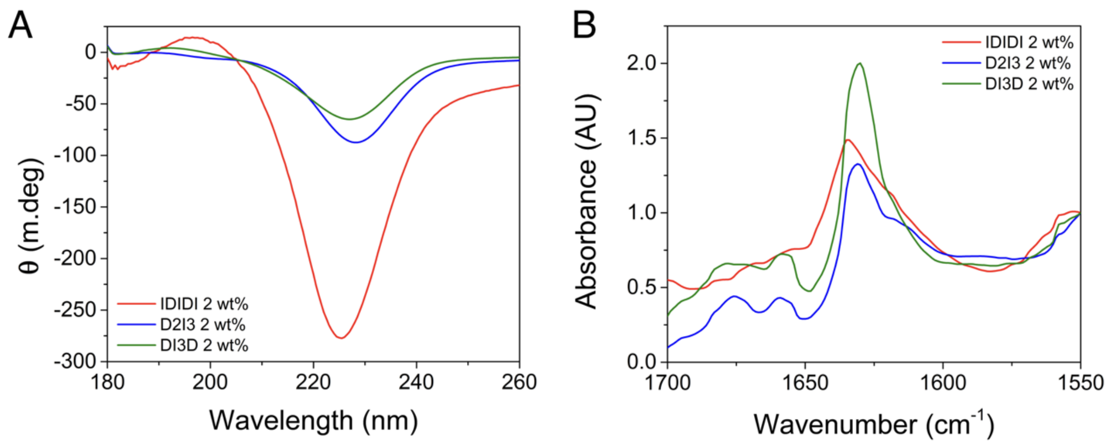

Figure S7. Spectroscopic studies of the pentapeptide hydrogels at 2 wt%. A) Circular dichroism showing minima for hydrogels between 220 nm and 230 nm, typically indicative of β-sheet formation B) FTIR spectra of the amide I region, with a peak at 1630 cm^{-1} indicating β-sheet formation.

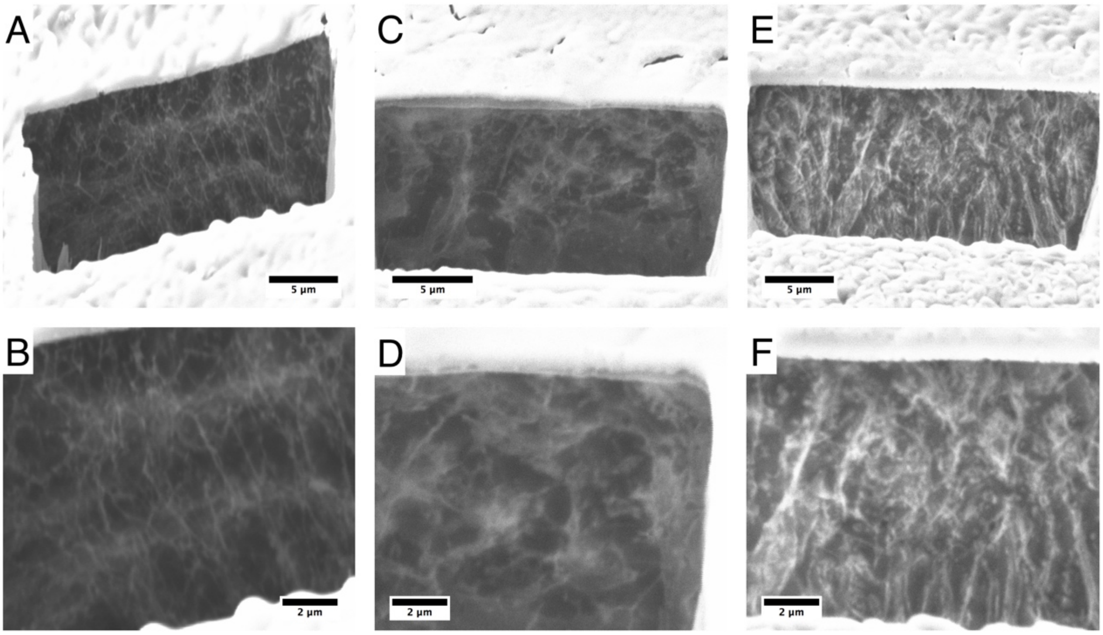

Figure S8. Cryo-FIB SEM images of 1 wt% hydrogels. A&B) IDIDI, C&D) D2I3 and E&F) DI3D.

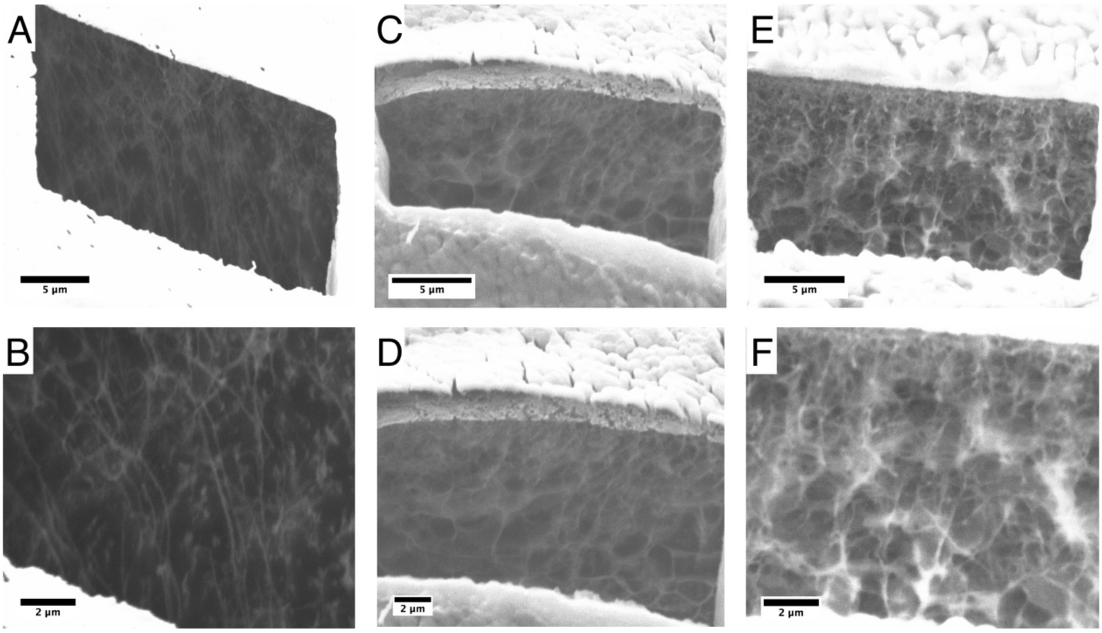

Figure S9. Cryo-FIB SEM images of 2 wt% hydrogels A&B) IDIDI, C&D) D2I3 and E&F) DI3D.

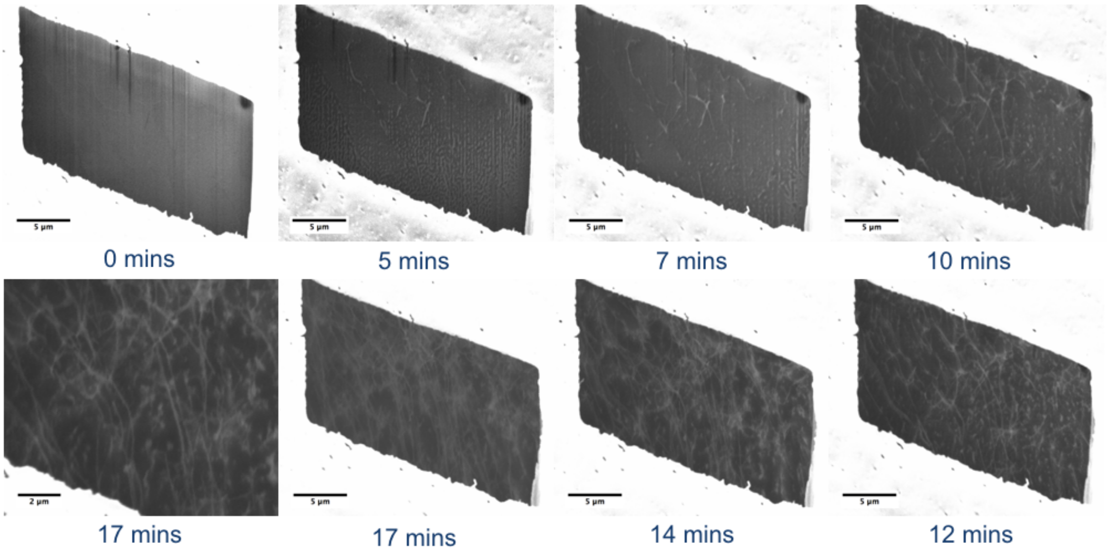

Figure S10. Cryo-FIB SEM images of 2 wt% IDIDI hydrogels as water is sublimed from the gel-water interface, revealing the hydrogel's underlying physical structure.

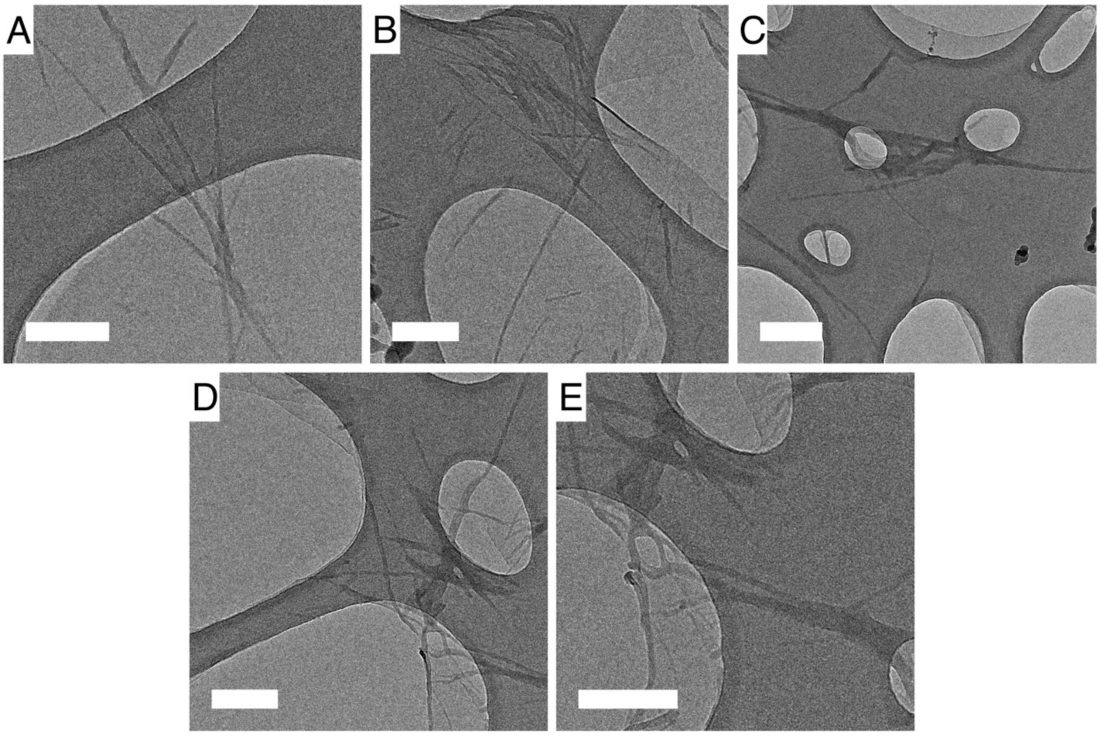

Figure S11. Cryo-TEM images of 2 wt% D2I3 hydrogels, scale bar on all images is 200nm.

Experimental

Materials

All Fmoc-protected amino acids, solvents and rink amide 4-methyl-benzhydrylamine (MBHA) resin used for peptide synthesis were purchased from AGTC Bioproducts (UK). All other solvents and reagents were purchased from Sigma-Aldrich (UK) and used as received.

Peptide Synthesis and Characterisation

All peptide sequences were synthesized using solid-phase methodology (FMOC, tBu, MBHA resin) on an automated microwave peptide synthesiser (Liberty, CEM). Crude Peptides were cleaved from the resin with a cleavage cocktail of 95% trifluoroacetic acid, 2.5% triisopropyl silane and 2.5% DI H_2O and left to shake for 2.5 h. Following cleavage, the crude peptides were precipitated and washed with cold diethyl ether (DEE), then left to dry under vacuum overnight.

The crude peptides were then purified by high pressure liquid chromatography (HPLC) using a Phenomenex C18 Kinetic-Evo column with a 5 micron pore size, a 110 Å particle size and with the dimensions 150 x 21.2 mm. A gradient from 5% acetonitrile 95% water to 100% acetonitrile was run with 0.1% ammonium hydroxide and 20 mM ammonium formate to prevent aggregation. Following purification, peptide identities were verified by analytical HPLC and accurate mass electronspray ionization mass spectrometry (ESI-MS) (Figure S1).

Hydrogel Preparation

For all experiments, individual hydrogel samples were prepared by first suspending the peptides in a 0.1M NH_4OH solution (pH 10) at a concentration of 1 or 2 wt% and then sonicating for 30 min until completely dissolved. From this stock solution, 35 ul aliquots were pipetted onto a hydrophobic surface and 5 ul of HCl (0.8 M) added to each droplet, resulting in immediate gelation. The individual gel samples were then sealed in 1.5 ml eppendorf centrifuge containers and left to equilibrate for 30 min, before verifying pH = 7.

Rheology

All rheological studies were performed on a TA-rheometer using 40 ul gel samples, a 8 mm parallel plate and a 0.5 mm gap distance at 25 °C. Before each experiment a 20 min soak period was applied for each sample. Frequency sweeps were performed at 0.1% strain and at oscillation frequencies from 0.1 – 100 rad s^{-1}. Strain sweeps had a fixed oscillation frequency of 6.283 rad s^{-1} and variable applied strain of 0.01 – 200%. Flow sweeps operated a shear rate from 0.1 – 100 with a 5s equilibration time and 30s averaging time.

For the recovery experiments, a step-time procedure was utilised with a series of applied strains. Initially the samples were to 0.1% applied strain for 30 s, followed immediately by a 200% applied strain for 30 s and finally, a 0.1% applied strain for 45 min. The same procedure was followed for the repeated recovery experiments (Figure S4) except a recovery time of 10 minutes was utilised and this was cycled 3 times. All steps were performed at a fixed oscillation frequency of 6.283 rad s^{-1}.

Circular Dichroism

Circular Dichroism (CD) measurements were carried out in the far-UV on a Chirascan spectrometer (Applied Photophysics). All scans were performed at 25°C from 180-260 nm at 0.5 nm intervals with an acquisition time of 1 second and using a 0.1mm path length quartz plate cuvette. The spectra of the hydrogels were taken after a 30 min equilibrium period, averaged across two scans and corrected via a background subtraction.

Fourier Transform Infrared Spectroscopy

To further probe the formation of β-sheet structures in the hydrogels, Fourier transform infrared spectroscopy (FT-IR) was carried out using a Perkin-Elmer Spectrum 100 FT-IR Spectrometer in the amide I region (1550 - 1700 cm^{-1}) at a resolution of 1 cm^{-1} and averaged from 8 consecutive scans. The hydrogels were lyophilized, and the resultant dried material used for analysis. All the spectra were normalized to the absorbance at 1550 cm^{-1}, which is assumed to be insensitive to peptide secondary structure.

Cryo-Focused Ion Beam Scanning Electron Microscopy

Cryo-Focused Ion Beam Scanning Electron Microscopy (cryo-FIB SEM) was carried out using a FEI Quanta 200 3D microscope fitted with a Quorum 3010T cryo-stage and preparation chamber. Samples were prepared for analysis by plunge freezing into liquid ethane on 12 mm aluminium disks, using a Gatan CP3 freezing system. The disks were then secured onto the sample shuttle of the cryo-system before transfer to the preparation station at -170 °C) via a liquid nitrogen slushing pot. The gels were sputtered in an argon environment using platinum for 60 s at a current of 10 mA. Once coated the shuttle was transferred to the cryo-stage in the SEM chamber and maintained at -170°C. Prior to cryo-FIB milling the surface of the samples was prepared by the deposition of an organometallic precursor layer dispensed from the gas injection system (GIS) of the microscope. The GIS crucible is warmed to 27 °C and the sample stage moved to be approximately 1 – 1.5 mm below the GIS needle (once inserted). The layer of the organometallic precursor was deposited for 10 s. This prepared layer was then milled using a FIB of gallium ions at currents of 3 nA – 0.1 nA to generate a cross-section in the sample with an exposed featureless face. To observe the structure of the gels, water is removed from the surface of this face by increasing the stage temperature to -100 °C and held for a period of 30 min (controlled by the software of the cryo-system). During this time, water sublimes away from the gel-water interface and through topological differences, the physical structure of the hydrogel is revealed. Finally, the microstructure of the hydrogel can then be imaged by the SEM at an accelerating voltage of 10 kV (Figures S8 & 9).

We have provided an example of the sublimination process, where bound water at the surface is removed, revealing the hydrogel's underlying physical structure (Figure S10). This technique allows for imaging of the hydrogels in their native state and in the presence of bound water, overcoming major artefacts associated with drying and water removal.

Cryo-Transmission Electron Microscopy

Cryo-Transmission Electron Microscopy (cryo-TEM) measurements were performed on a JEOL 2100+ TEM using a Gatan 626 cryo-TEM holder. Copper TEM grids (Holey carbon on 300 mesh Cu) were glow discharged for 10 seconds at 5 mA using an Agar turbo coater aux. power unit and a dedicated glow discharge head. Samples

were prepared for analysis by placing 12 ul of the D2I3 2 wt% hydrogel sample onto the prepared TEM grid, blotting for 2-5 seconds and then plunge freezing into liquid ethane, using a Gatan CP3 freezing system. Samples were then transferred in cryo-TEM storage boxes before loading onto the cryo-TEM holder. The images were acquired for 2-4 seconds at a dose of below 10 e/A^2 using a US1000 CCD camera and Digital Micrograph GMS 3.
